# Dietary Determinants of Mental Well-Being Among Cardiometabolic High-Risk Adults in Hungary

**DOI:** 10.3390/nu18132086

**Published:** 2026-06-26

**Authors:** Battamir Ulambayar, Bashar Shehab, Attila Sárváry, Attila Csaba Nagy

**Affiliations:** 1 Department of Epidemiology, Faculty of Health Sciences, University of Debrecen, 4032 Debrecen, Hungary; ulambayar.battamir@etk.unideb.hu; 2 Department of Nursing and Integrative Health Sciences, Institute of Health Sciences, Faculty of Health Sciences, University of Debrecen, 4400 Nyiregyhaza, Hungarysarvary.attila@etk.unideb.hu (A.S.)

**Keywords:** cardiometabolic risk, mental well-being, dietary habits, WHO-5, Hungary, European health interview survey

## Abstract

**Background**: Mental well-being is an important yet often overlooked component of cardiometabolic health. Dietary habits may influence psychological outcomes, but evidence among high-risk populations in Central and Eastern Europe remains limited. This study investigated the association between dietary behaviors and mental well-being among adults with cardiometabolic risk in Hungary. **Methods**: A cross-sectional analysis was conducted using data from the European Health Interview Survey (EHIS) 2019. The study included 2785 adults with cardiometabolic high risk (obesity, hypertension, or hypercholesterolemia). Mental well-being was assessed using the WHO-5 Well-Being Index and categorized as poor (≤50) or better (>50). Dietary habits, sociodemographic factors, and lifestyle factors were analyzed. Weighted multivariable logistic regression was used to estimate adjusted odds ratios (ORs) and 95% confidence intervals (CIs). **Results**: Overall, 25.9% of participants had poor mental health. In multivariable analyses, low intake of vegetables (OR = 1.15), fruits (OR = 1.55), fruit juice (OR = 1.26), and fish (OR = 1.17), as well as inadequate water intake (OR = 1.38), were each independently associated with higher odds of poor mental health after adjustment for sex, education, income levels, self-perceived health status, physical activity, and alcohol consumption. **Conclusions**: Healthier dietary behaviors, particularly higher consumption of vegetables, fish, and adequate hydration, are associated with better mental well-being among individuals with cardiometabolic risk. These results underscore the need for comprehensive intervention strategies that simultaneously address physical health and psychological well-being among vulnerable populations.

## 1. Introduction

Cardiometabolic conditions refer to a cluster of interrelated disorders, including obesity, hypertension, and dyslipidemia, that share common underlying mechanisms such as insulin resistance and chronic inflammation [1]. Based on estimates from the 2019 Global Burden of Disease (GBD) Study, cardiometabolic risk factors such as high systolic blood pressure and high body mass index remain leading contributors to global mortality and disability, with high blood pressure alone accounting for approximately 10.8 million deaths, which is 19.2% of worldwide mortality [2]. Central and Eastern Europe continues to experience a considerable disease burden, largely driven by persistently high rates of obesity and hypertension [3]. Hungary is among the most affected countries, with high rates of cardiometabolic risk factors contributing substantially to national morbidity and mortality [4].

Mental health is an important but often underrecognized component of cardiometabolic health. Individuals with cardiometabolic conditions such as obesity, hypertension, and dyslipidemia are at increased risk of poor mental well-being, including depression and reduced quality of life [5]. This relationship is bidirectional, as mental health disorders can adversely affect health behaviors, treatment adherence, and disease progression, while the burden of chronic physical conditions may negatively impact psychological well-being [6]. Better mental health in this population or patients who received mental healthcare has been associated with better clinical outcomes, increased healthcare utilization, and lower mortality [7]. Therefore, understanding factors associated with mental well-being among individuals with cardiometabolic risk is crucial for developing comprehensive intervention and prevention strategies.

Dietary patterns have been increasingly recognized as important determinants of mental health, particularly among individuals with cardiometabolic conditions. Diets rich in fruits, vegetables, fish, and whole foods are associated with better mental well-being, potentially through mechanisms involving reduced inflammation, improved metabolic regulation, and modulation of the gut–brain axis [8]. In contrast, poor dietary habits, including low intake of nutrient-dense foods and inadequate hydration, may contribute to worse psychological outcomes [8]. Among individuals with cardiometabolic risk, diet plays a dual role by influencing both physical and mental health, making it a key target for integrated prevention strategies [9].

Physical activity is another important behavioral determinant of mental well-being, with regular activity associated with reduced depression and anxiety through neurobiological mechanisms including neurotrophic factor expression, hypothalamic–pituitary–adrenal axis modulation, and systemic inflammation reduction [10]. Sedentary behavior is disproportionately prevalent among individuals with cardiometabolic risk and has been independently linked to poorer mental health outcomes [11]. Dietary habits and physical activity tend to cluster within individuals, share common socioeconomic determinants, and may interact synergistically in their effects on psychological well-being [12]. Yet most studies have treated physical activity merely as a covariate rather than as a potential modifier of dietary associations, leaving the joint influence of these behaviors on mental well-being insufficiently examined in cardiometabolic high-risk populations.

Despite growing evidence on the role of diet in mental health, most studies have focused on the general population, with relatively limited attention given to individuals with cardiometabolic risk. This population may have unique vulnerabilities due to the coexistence of metabolic disturbances and lifestyle-related risk factors. Moreover, socioeconomic determinants, such as education and income, may further influence both dietary habits and mental well-being, potentially confounding observed associations [13]. Understanding these interrelationships is particularly important in countries like Hungary, where both cardiometabolic burden and health inequalities are pronounced.

Existing studies have often focused on single dietary components rather than examining multiple dietary behaviors and lifestyle factors simultaneously, potentially overlooking their combined and interactive effects on mental health outcomes. Moreover, although the relationship between diet and mental health has been widely investigated in general populations, evidence specifically addressing individuals with clustered cardiometabolic risk factors remains limited. This gap is particularly evident in Central and Eastern Europe, where population-based studies using standardized and internationally comparable data sources, such as the European Health Interview Survey (EHIS), are scarce, limiting the regional evidence base and the comparability of findings across populations.

Therefore, this study aimed to investigate the associations between dietary habits and mental well-being among adults with cardiometabolic high risk in Hungary using EHIS 2019 data, while also examining the role of sociodemographic and lifestyle factors and exploring potential interaction effects between dietary behaviors and physical activity on mental well-being outcomes.

## 2. Methodology

### 2.1. Research Design and Data

Data for the present cross-sectional analysis were obtained from the Hungarian implementation of the EHIS 2019, which represents the latest available survey cycle. The EHIS is a Europe-wide health monitoring initiative organized under the coordination of Eurostat and designed to generate comparable health-related data across participating countries. In Hungary, the survey was administered by the Hungarian Central Statistical Office during 2019. A nationally representative sample of individuals aged 15 years and older living in private households was selected through a stratified, multistage sampling framework. Stratification was based on regional and settlement-level characteristics to ensure adequate geographic coverage. Sampling proceeded by first selecting households and subsequently choosing one eligible household member at random for participation.

To obtain population-level estimates, all statistical analyses incorporated the survey weights supplied with the EHIS dataset. These weights adjust for differences in selection probabilities and compensate for non-response, thereby improving the representativeness of the findings for the Hungarian population. Information was gathered using a combination of interviewer-administered surveys and self-completed questionnaires. The dataset contains extensive information on demographic and socioeconomic characteristics, health conditions, health-related behaviors, dietary practices, and psychological well-being [14].

For the present study, the analytical sample was restricted to participants classified as having cardiometabolic high risk, defined as the presence of at least two of the following conditions: obesity, hypertension, or hypercholesterolemia. Participants were classified as having elevated cardiometabolic risk if they presented at least two of the following three conditions: obesity, hypertension, and hypercholesterolemia. Obesity was defined as a body mass index (BMI) ≥ 30 kg/m^2^. Hypertension was defined as self-reported physician-diagnosed hypertension. Hypercholesterolemia was defined as self-reported physician-diagnosed high blood cholesterol. The requirement of at least two concurrent cardiometabolic abnormalities was used to identify individuals with a higher burden of cardiometabolic risk factors. This approach is based on the concept of cardiovascular risk factor clustering, whereby the coexistence of multiple metabolic abnormalities is associated with substantially increased cardiovascular morbidity and mortality compared with isolated risk factors [15]. Individuals with a history of stroke, acute myocardial infarction, or coronary artery disease were excluded. Participants were included if they had complete data on mental health status (WHO-5), dietary variables, and relevant covariates.

### 2.2. Variables

The primary outcome was mental well-being, assessed using the World Health Organization-Five Well-Being Index (WHO-5). Scores range from 0 to 100, with higher scores indicating better mental well-being. Consistent with established cut-offs, the outcome was dichotomized as poor mental health (≤50) and better mental well-being (>50) [16].

Information on food consumption patterns was derived from the EHIS nutrition questionnaire, which records how often participants consume a range of commonly eaten foods and drinks. Variables included vegetable consumption, fruit consumption, fruit juice intake, water intake, fish and seafood consumption, dairy products, meat, sweets, salt use, coffee or tea consumption, and sweetener use. These variables were categorized based on the reported frequency of intake.

Sociodemographic and lifestyle variables were included as covariates based on their known associations with mental health. These included sex, age group, educational attainment, household income quintiles, self-perceived health status, and physical activity related to main daily activity.

### 2.3. Statistical Analysis

Participant characteristics were summarized using descriptive analytical methods. Categorical data are reported as counts and corresponding proportions. Associations between mental health status and participant characteristics, including dietary factors, were examined using Pearson’s chi-square test.

Given that mental health status was categorized as a dichotomous outcome, a binary logistic regression model was fitted to identify factors independently associated with poor mental well-being. Sociodemographic characteristics, lifestyle behaviors, and dietary variables were examined in bivariate analyses. Variables demonstrating significant associations in bivariate analyses, together with variables considered clinically and theoretically relevant based on previous literature, were included in the final multivariable model. For the multivariable analysis, dietary variables were retained in their original frequency categories where possible; categories with very low frequencies were merged where necessary to ensure model stability and reduce sparse-data bias. For fish consumption, the two highest intake categories were combined into a single ‘more than once per week’ reference group due to the small number of participants in the highest frequency category. All other dietary variables retained their original three-level frequency categories as reported in the EHIS questionnaire. The results are presented as adjusted odds ratios (ORs) with 95% confidence intervals (CIs), where ORs greater than 1 indicate higher odds of poor mental health and ORs less than 1 indicate lower odds compared with the reference category. Multicollinearity among independent variables was assessed using variance inflation factors (VIFs). No evidence of problematic multicollinearity was observed, as all VIF values were below commonly accepted thresholds.

To examine whether physical activity modified the association between dietary behaviors and mental well-being, interaction terms between physical activity level and each dietary variable (vegetable, fruit, fruit juice, water, and fish consumption) were introduced into separate multivariable logistic regression models. The overall statistical significance of each interaction was assessed using the Wald test.

All estimates incorporated the sampling weights provided with the survey dataset, thereby accounting for the survey design and enhancing the generalizability of the findings to the Hungarian population. The predictive performance of the regression models was assessed using the area under the receiver operating characteristic (ROC) curve. Statistical analyses were performed in Stata 18.0 (StataCorp LLC, College Station, TX, USA) [17].

### 2.4. Ethical Considerations

The present investigation utilized de-identified data from the EHIS 2019 and did not involve direct participant recruitment or data collection. Permission to use the dataset for research purposes was obtained from the Ethics Committee of the University of Debrecen (Reference No. 5609-2020; approved on 17 December 2020). All study processes met the terms of applicable legal requirements and institutional ethical principles. Participant consent had been secured during the original survey administration, and no further consent procedures were necessary for the current secondary data analysis.

## 3. Results

Out of a total of 5603 participants in the Hungarian EHIS 2019 dataset, 2785 individuals were identified as having cardiometabolic high risk and were included in the analysis. Based on WHO-5 scores, 721 participants (25.9%) were classified as having poor mental health, while 2064 (74.1%) had better mental well-being.

Table 1 presents the distribution of sociodemographic, lifestyle, and health characteristics by mental health status. Several factors showed statistically significant associations with poor mental well-being. Socioeconomic gradients were evident across both education and income, with higher prevalence of poor mental health among participants with lower educational attainment and those in the lowest income quintile (both *p* < 0.001). Sex differences were also observed, with females reporting poor mental health more frequently than males (*p* < 0.001). The strongest association was found for self-perceived health status, which showed a pronounced dose–response relationship: the proportion of poor mental health increased nearly eightfold from the very good to the very bad self-rated health category (*p* < 0.001). Physical activity demonstrated a consistent inverse association, with sedentary participants showing the highest prevalence of poor mental health across all activity levels (*p* < 0.001). Alcohol consumption showed a non-linear pattern, with moderate consumers having the lowest prevalence of poor mental health, while both non-drinkers and heavy consumers had comparatively higher proportions (*p* < 0.001). Age and smoking status were not significantly associated with mental health status.

Several dietary behaviors were significantly associated with mental health status in bivariate analyses (Table 2). A consistent pattern emerged across multiple food groups: participants with lower consumption frequency of vegetables, fruits, fruit juice, and fish, as well as those with inadequate daily water intake, had higher proportions of poor mental health compared to their more frequent-consuming counterparts. The strongest gradient was observed for fruit consumption (*p* < 0.001), followed by vegetable and fish intake, and hydration status (all *p* = 0.001). In contrast, consumption of dairy products, red meat, sweets, and salt did not show statistically significant associations with mental health status. These findings suggest that higher intake of nutrient-dense foods and adequate hydration are associated with better mental well-being, while energy-dense or processed food behaviors showed no clear association in this population.

In the multivariable logistic regression analysis (Table 3), several independent predictors emerged across demographic, health, and lifestyle domains. Among demographic factors, female sex was linked with modestly higher odds of poor mental health status (OR 1.40), while education and income showed no statistically significant gradient, suggesting that socioeconomic position may operate through pathways not captured in this model. Self-reported health status showed the strongest associations, exhibiting a pronounced dose–response relationship: odds increased progressively from “Good” to “Very bad,” with the poorest self-rated health category associated with nearly 15-fold higher odds compared to “Very good”, a pattern consistent with self-perceived health as a sensitive integrative marker of overall health status. Physical activity at work or in daily life was consistently protective across all active categories relative to sedentary behavior, with odds reductions of roughly 37–43%, and no meaningful difference in effect size between light, moderate, and heavy exertion levels. Alcohol consumption was independently associated with mental health status. Compared with heavy alcohol consumers, moderate alcohol consumers had 43% lower odds of poor mental health.

Dietary patterns revealed that infrequent consumption of vegetables, fruit, fruit juice, and water, as well as low fish intake, were each independently associated with higher odds of the outcome. The strongest dietary associations were observed for fruit and water consumption, where the least frequent intake categories carried 55% and 38% higher odds, respectively. Notably, fruit juice and fish showed more modest but statistically significant associations only at the lowest frequency categories, suggesting a threshold rather than linear effect. The final model demonstrated acceptable discriminatory ability, with an AUC of 0.735 (95% CI: 0.712–0.757).

Table 4 presents the results of the interaction analyses examining whether the associations between dietary behaviors and poor mental well-being varied according to physical activity level. Among sedentary individuals, low fruit consumption (1–3 times/week vs. daily) was associated with 67% higher odds of poor mental health (OR 1.67, 95% CI 1.22–2.29), whereas among moderately active individuals, daily fruit intake was independently protective (OR 0.64, 95% CI 0.47–0.87). Similarly, the protective association of regular vegetable and fish consumption with mental well-being was more pronounced among physically active participants. The association of inadequate water intake with poor mental health was particularly evident among sedentary individuals (OR 1.80, 95% CI 1.26–2.58).

## 4. Discussion

This study investigated the associations between dietary behaviors and mental well-being among adults with cardiometabolic high risk in Hungary using nationally representative EHIS 2019 data. Approximately one-quarter of participants experienced poor mental health, highlighting the substantial psychological burden within this vulnerable population. The findings showed that healthier dietary behaviors, particularly higher consumption of vegetables and fish, as well as adequate water intake, were independently associated with better mental well-being after adjustment for sociodemographic and lifestyle factors. In addition, female sex, poor self-perceived health, alcohol consumption and sedentary lifestyle were independently associated with higher odds of poor mental health.

The observed association between vegetable consumption and better mental well-being is consistent with previous evidence linking diets rich in plant-based foods to improved psychological outcomes [18]. Vegetables are major sources of vitamins, minerals, antioxidants, dietary fiber, and phytochemicals that may contribute to mental health through anti-inflammatory and neuroprotective pathways [19]. Chronic low-grade inflammation is recognized as a common underlying mechanism in both cardiometabolic diseases and depression, and diets rich in vegetables may help reduce inflammatory burden and oxidative stress [20]. Furthermore, dietary fiber may promote a more favorable gut microbiota composition and increase the production of short-chain fatty acids, which have been implicated in gut–brain signaling pathways involved in mood regulation [21]. The present findings support these mechanisms, suggesting that sufficient vegetable consumption may be associated with better mental well-being among individuals with cardiometabolic risk.

Low fruit consumption was independently associated with poorer mental well-being in the present study. Participants consuming fruit less than once per week had significantly higher odds of poor mental health compared with those consuming fruit more than four times per week, supporting previous evidence linking regular fruit intake to better psychological health [22]. Fruits provide a range of nutrients and bioactive compounds, including vitamins, antioxidants, polyphenols, and dietary fiber, which may contribute to mental well-being through anti-inflammatory, antioxidant, and gut microbiota-related mechanisms [23].

The association observed for fruit juice consumption should be interpreted more cautiously. Although participants consuming fruit juice less than once per week also exhibited higher odds of poor mental health, the magnitude of the association was smaller and only marginally significant after adjustment. Fruit juice consumption may reflect broader dietary habits and lifestyle characteristics that were not fully captured in the analysis. Moreover, individuals who consume fruit juice regularly may also be more likely to engage in other health-promoting behaviors [24]. Because fruit and fruit juice consumption represent related dietary behaviors and were included simultaneously in the multivariable model, some degree of correlation between these variables may have influenced the individual effect estimates. Nevertheless, the persistence of associations for both variables suggests that dietary patterns characterized by low consumption of fruit-based foods and beverages may be linked to poorer mental well-being among adults with elevated cardiometabolic risk.

The observed associations between lower consumption of vegetables and fruit-related products and poorer mental health should also be interpreted within the Hungarian dietary context. Previous studies have shown that Hungary remains below recommended fruit and vegetable intake levels, with substantial socioeconomic differences in consumption patterns. Compared with Mediterranean countries, vegetable and fruit consumption is generally lower in Central and Eastern Europe, which may contribute to the higher burden of cardiometabolic diseases observed in the region [25]. Therefore, regular consumption of plant-based foods may be an important correlate of both physical and mental health among Hungarian adults at elevated cardiometabolic risk.

Fish consumption was also independently associated with better mental well-being. This finding aligns with prior studies demonstrating beneficial effects of fish and seafood intake on depression and psychological well-being, largely attributed to the high content of omega-3 polyunsaturated fatty acids [26,27]. Omega-3 fatty acids may influence neurotransmitter signaling, neuroinflammation, and neuronal membrane function, thereby contributing to improved emotional regulation and cognitive health [28]. In cardiometabolic high-risk populations, where inflammatory and metabolic dysregulation are common, regular fish consumption has been associated with favorable cardiovascular and mental health outcomes in previous studies [29]. However, fish consumption in Hungary and several Central and Eastern European countries remains relatively low compared with Western European populations [30]. Low fish consumption in Hungary has been attributed to dietary traditions, limited habitual fish intake, and product-related barriers, including the presence of fishbones, perceived inconsistencies in quality, and the limited availability of convenient ready-to-cook or ready-to-eat fish products [31]. Consequently, even modest increases in fish consumption may represent an important and achievable public health target in Hungary and other Central and Eastern European countries with similarly low baseline consumption levels.

Adequate hydration was another important factor associated with better mental well-being in the present study. Participants consuming less than one liter of water per day had significantly higher odds of poor mental health compared with those consuming more than two liters daily. Although hydration is often overlooked in mental health research, adequate fluid intake is essential for normal brain function. Even mild dehydration has been associated with impaired attention, reduced cognitive performance, increased fatigue, and mood disturbances [32]. These effects may be mediated through physiological mechanisms involving electrolyte imbalance, hormonal responses, altered cerebral perfusion, and disruptions in neurocognitive functioning. Consequently, insufficient water intake may adversely affect psychological well-being and contribute to poorer mental health outcomes [33]. Individuals with cardiometabolic conditions may be particularly vulnerable to the adverse effects of inadequate hydration because of concurrent metabolic disturbances and medication use [34,35]. These findings emphasize the importance of considering hydration as part of holistic lifestyle recommendations for cardiometabolic high-risk populations.

Several dietary variables, including dairy product consumption, red meat consumption, sweets intake, and salt-related dietary behaviors, which have previously been suggested to influence mental health [36,37,38,39], were not independently associated with poor mental health in the adjusted analyses. This may reflect small effect sizes, limitations of self-reported dietary frequency measures, or the influence of broader dietary patterns and lifestyle factors that were not fully captured in the present study.

The study also identified strong associations between non-dietary factors and mental health. Female participants had higher odds of poor mental health than males, which is consistent with the existing literature showing a higher prevalence of depressive symptoms and psychological distress among women [40]. Biological, hormonal, psychosocial, and caregiving-related factors may contribute to this disparity [41]. Self-perceived health status showed the strongest association with mental well-being, with markedly increasing odds of poor mental health among participants reporting poorer health [42,43]. This finding highlights the close interrelationship between physical and psychological health in individuals with cardiometabolic risk.

Similarly, physical activity demonstrated a protective association against poor mental health, supporting previous evidence that active lifestyles improve mood, reduce stress, and enhance overall well-being through both physiological and psychosocial mechanisms [44,45]. The interaction analyses revealed that physical activity significantly modified the association between dietary behaviors and mental well-being across the significant dietary factors examined. Sedentary individuals with low fruit, vegetable, and water intake appeared to be at the greatest risk of poor mental health, while the mental health benefits of healthier dietary behaviors were more consistently observed among physically active participants. These findings suggest that diet and physical activity may act synergistically on mental well-being, rather than through fully independent pathways [46]. In the context of cardiometabolic high-risk populations, where both sedentary behavior and poor dietary habits tend to co-occur, these results highlight the potential value of integrated lifestyle interventions that simultaneously target dietary quality and physical activity. Such combined approaches may be more effective than single-domain strategies in supporting mental well-being in this vulnerable group.

The finding that moderate alcohol consumers had lower odds of poor mental health than heavy drinkers is consistent with previous studies reporting better psychological well-being among moderate drinkers [47,48]. This association may partly reflect greater social engagement and healthier lifestyle characteristics. In addition, moderate alcohol consumption has been associated with a lower risk of cardiovascular disease in some observational studies [49,50], which may indirectly contribute to better overall well-being. However, given the known adverse health effects of alcohol and the potential for residual confounding, these findings should not be interpreted as evidence of a protective effect of alcohol consumption itself.

From a public health perspective, the findings suggest that interventions targeting individuals with elevated cardiometabolic risk should promote practical and achievable dietary behaviors, including daily consumption of fruits and vegetables, regular fish consumption, and adequate water intake. Given the relatively low fish and vegetable consumption observed in Hungary, low-cost strategies such as nutrition education campaigns, workplace health promotion programs, community-based interventions, and initiatives aimed at improving access to affordable healthy foods may represent feasible approaches to support both physical and mental health.

Several limitations should be acknowledged. First, the cross-sectional design prevents conclusions regarding causality or temporal relationships. Consequently, the direction of the observed associations cannot be determined, and reverse causation cannot be excluded. Poor mental health may influence dietary behaviors, while dietary habits may also be associated with mental well-being, suggesting the possibility of bidirectional relationships. Second, dietary intake and health-related variables were self-reported and therefore subject to recall bias and reporting inaccuracies. Third, the assessment of dietary habits using frequency-based measures only. The EHIS does not capture portion sizes, total food consumption, nutrient composition, or overall dietary patterns. Consequently, participants with similar consumption frequencies may differ substantially in actual intake, which could result in dietary misclassification and attenuate observed associations. Future studies should incorporate more comprehensive dietary assessment methods, such as validated food frequency questionnaires, dietary recalls, or dietary pattern analyses, to better characterize the relationship between dietary behaviors and mental well-being. Fourth, the possibility of residual confounding due to unmeasured factors, such as sleep quality, medication use, social support, or psychiatric history, cannot be ruled out. Finally, because the WHO-5 measures subjective well-being rather than clinically diagnosed psychiatric disorders, the results should not be interpreted as reflecting specific mental health conditions.

However, the interpretation of the findings is strengthened by several key attributes of the study design. These include access to a large representative population sample, consistent survey administration procedures, the use of a psychometrically established measure of mental well-being, and adjustment for a range of demographic and behavioral characteristics that may have affected the results.

## 5. Conclusions

In this cross-sectional study of adults with cardiometabolic high risk in Hungary, several dietary and lifestyle factors were associated with mental well-being. Specifically, lower consumption of vegetables, fruits and fish, inadequate hydration, and reduced physical activity were independently associated with higher odds of poor mental well-being, while better self-perceived health showed a strong and graded inverse association with poor psychological outcomes.

These findings are associative in nature and do not permit causal inference. The cross-sectional design precludes conclusions regarding the direction or temporality of the observed relationships, and reverse causation cannot be excluded. Nevertheless, the consistency of findings across multiple dietary and lifestyle domains suggests that integrated prevention strategies addressing both physical and mental health may be relevant for this population.

Given that poor dietary behaviors, physical inactivity, and poor mental well-being tend to co-occur among individuals with cardiometabolic risk, single-domain interventions targeting only diet or only physical activity may be insufficient. The significant interactions observed between physical activity and dietary behaviors suggest that integrated lifestyle interventions addressing both domains simultaneously may offer greater benefits for mental well-being than approaches targeting either factor alone. In Hungary and similar Central and Eastern European settings, where cardiometabolic burden is high and both vegetable and fish consumption remain below recommended levels, practical strategies such as community-based nutrition education, workplace health promotion programs, and initiatives improving access to affordable healthy foods warrant consideration. These approaches should be designed to reach socioeconomically disadvantaged groups, who face the greatest barriers to healthy dietary behaviors and who may be disproportionately affected by poor mental well-being. Ultimately, integrating mental health promotion into cardiometabolic prevention strategies, rather than treating them as separate priorities, may represent a more effective and efficient use of public health resources. Longitudinal and interventional studies are needed to determine whether these associations reflect causal relationships and to evaluate the potential impact of integrated dietary and physical activity interventions on mental well-being in individuals with cardiometabolic risk.

## Figures and Tables

**Table 1:** Characteristics of the study population according to mental health status.

Variables	Category	Mental Well-Being (WHO-5)		*p*-Value *
		Poor (*n*, %)	Better (*n*, %)	
Sex	Male	266 (21.4)	979 (78.6)	**<0.001**
	Female	455 (29.6)	1085 (70.4)	
Age group	18–34	54 (25.8)	155 (74.2)	0.928
	35–64	338 (25.6)	984 (74.4)	
	≥65	329 (26.2)	925 (73.28)	
Education	Primary	233 (36.5)	406 (63.5)	**<0.001**
	Secondary	381 (23.4)	1246 (76.6)	
	Higher	107 (20.6)	412 (79.4)	
Income level	Low (1st)	198 (33.1)	401 (66.9)	**<0.001**
	Middle low (2nd)	179 (26.6)	493 (73.4)	
	Middle (3rd)	138 (23.7)	444 (76.3)	
	Middle high (4th)	132 (21.8)	474 (78.2)	
	High (5th)	74 (22.7)	252 (77.3)	
Self-reported health status	Very good (ref)	14 (8.2)	158 (91.8)	**<0.001**
	Good	122 (13.8)	763 (86.2)	
	Satisfactory	314 (25.5)	918 (74.5)	
	Bad	196 (52.1)	180 (47.9)	
	Very bad	68 (62.4)	41 (37.6)	
Physical demands of work or main daily activity	Sedentary or inactive	353 (33.9)	689 (66.1)	**<0.001**
	Light activity	65 (23.2)	215 (76.8)	
	Moderate activity	270 (21.1)	1015 (78.9)	
	Vigorous activity	26 (17.8)	120 (82.2)	
Smoking status	Active smoking	165 (26.4)	459 (73.6)	0.684
	Former smoking	161 (24.5)	495 (75.5)	
	No smoking	386 (26.1)	1091 (73.9)	
Alcohol consumptions	Heavy consumptions	40 (28.0)	103 (72.0)	**<0.001**
	Moderate consumptions	109 (18.8)	471 (81.2)	
	Rare consumptions	268 (24.4)	832 (75.6)	
	No alcohol consumptions	298 (31.6)	644 (68.4)	

* Statistically significant findings (*p* < 0.05) are highlighted in bold type and were identified using Pearson’s chi-square test.

**Table 2:** Associations between eating habits and mental health status.

Variables	Category	Mental Well-Being (WHO-5)		*p*-Value *
		Poor (≤50)	Better (>50)	
Vegetable consumptions	4 or more times a week	420 (23.9%)	1338 (76.1%)	**0.001**
	1–3 times a week	226 (28.1)	579 (71.9)	
	Less than once a week	73 (35.3)	134 (64.7)	
Fruit consumptions	4 or more times a week	280 (21.3)	1033 (78.7)	**<0.001**
	1–3 times a week	359 (29.6)	854 (70.4)	
	Less than once a week	77 (32.2)	162 (67.8)	
Fruit juice consumptions	4 or more times a week	40 (19.7)	163 (80.3)	**0.001**
	1–3 times a week	125 (21.6)	453 (78.4)	
	Less than once a week	550 (27.9)	1420 (72.1)	
Drinking water a day	More than 2 L a day	304 (22.7)	1034 (77.3)	**0.001**
	1–1.5 L a day	219 (27.6)	573 (72.4)	
	0.5–1 L a day	127 (29.4)	305 (70.6)	
	Less than 0.5	70 (32.6)	145 (67.4)	
Coffee or tea consumptions	3 or more times a day	178 (25.5)	521 (74.5)	0.667
	1–2 times a day	471 (25.8)	1353 (74.2)	
	Less than once a day	71 (28.3)	180 (71.7)	
Sweetener use for hot drinks	Natural sweetener	376 (25.6)	1091 (74.4)	0.917
	Artificial sweetener	157 (26.3)	439 (73.7)	
	No sweetener	115 (25.3)	340 (74.7)	
Consumptions of sweets and desserts	More than 3 portions a day	31 (22.3)	108 (77.7)	0.146
	1–3 portions a day	278 (24.4)	860 (75.6)	
	Less than once a day	408 (27.4)	1084 (72.6)	
Consumptions of dairy products	4 or more times a week	451 (23.4)	1328 (74.6)	0.120
	1–3 times a week	179 (25.3)	529 (74.7)	
	Less than once a week	88 (31.0)	196 (69.0)	
Salt use	Low	428 (25.6)	1399 (74.4)	0.598
	Moderate	200 (27.2)	536 (72.8)	
	High	35 (23.8)	112 (76.2)	
Red meat consumptions	4 or more times a week	79 (23.5)	257 (76.5)	0.09
	1–3 times a week	426 (25.1)	1273 (74.9)	
	Less than once a week	210 (28.8)	519 (71.2)	
White meat consumptions	4 or more times a week	137 (23.8)	439 (76.2)	0.128
	1–3 times a week	534 (26.0)	1517 (74.0)	
	Less than once a week	46 (31.9)	98 (68.1)	
Fish and seafood consumptions	4 or more times a week	10 (19.6)	41 (80.4)	**0.001**
	1–3 times a week	135 (20.6)	522 (79.4)	
	Less than once a week	570 (27.8)	1484 (72.2)	

* Statistically significant findings (*p* < 0.05) are highlighted in bold and were identified using Pearson’s chi-square test.

**Table 3:** Multivariable analysis of dietary determinants of poor mental well-being.

Variables	Category	OR	95% CI	*p*-Value *
Sex	Male (ref)			
	Female	1.40	1.14–1.71	**0.001**
Educational attainment	Primary (ref)			
	Secondary	0.93	0.73–1.18	0.557
	Higher	0.73	0.51–1.03	0.08
Income levels (EU quintiles)	Low (1st)			
	Middle low (2nd)	0.89	0.68–1.17	0.420
	Middle (3rd)	0.91	0.68–1.23	0.559
	Middle high (4th)	0.93	0.68–1.26	0.689
	High (5th)	1.30	0.88–1.91	0.181
Self-reported health status	Very good (ref)			
	Good	1.86	1.01–3.41	**0.044**
	Satisfactory	3.92	2.17–7.08	**<0.001**
	Bad	11.69	6.03–21.69	**<0.001**
	Very bad	14.58	7.13–29.78	**<0.001**
Physical demands of work or main daily activity	Sedentary or inactive			
	Light activity	0.63	0.45–0.89	**0.009**
	Moderate activity	0.57	0.46–0.71	**<0.001**
	Vigorous activity	0.58	0.34–0.94	**0.029**
Alcohol consumptions	Heavy consumptions (Ref)			
	Moderate consumptions	0.57	0.36–0.91	**0.019**
	Rare consumptions	0.70	0.45–1.09	0.115
	No alcohol consumptions	0.67	0.42–1.06	0.08
Vegetable consumptions	4 or more times a week (Ref)			
	1–3 times a week	0.75	0.51–1.10	0.151
	Less than once a week	1.15	1.02–1.36	**0.036**
Fruit consumptions	4 or more times a week (Ref)			
	1–3 times a week	1.33	0.92–1.92	0.126
	Less than once a week	1.55	1.25–1.93	**<0.001**
Fruit juice consumptions	4 or more times a week (Ref)			
	1–3 times a week	0.98	0.63–1.53	0.958
	Less than once a week	1.26	1.03–1.36	**0.048**
Drinking water	More than 2 L a day (Ref)			
	1–1.5 L a day	1.20	0.96–1.53	0.105
	Less than 1 L a day	1.38	1.09–1.75	**0.006**
Fish consumptions	More than once a week (ref)			
	One or less a week	1.17	1.03–1.48	**0.034**

* Bold values indicate statistical significance (*p* < 0.05). Odds ratios (ORs) are adjusted for variables in the model. Overall associations for categorical dietary variables were confirmed using Wald tests (all *p* < 0.05). AUC = 0.735 (95% CI 0.712–0.757).

**Table 4:** Summary of interaction effects between physical activity level and dietary behaviors on poor mental well-being.

Dietary Variable	Wald χ^2^ (df)	*p*-Value	Most Vulnerable Combination	OR (95% CI)	*p*
Vegetable consumption	37.65 (11)	0.0001	Sedentary + <1×/week	0.84 (0.46–1.52)	0.555
Fruit consumption	49.40 (11)	<0.0001	Sedentary + 1–3×/week	1.67 (1.22–2.29)	0.001
Water intake	47.70 (11)	<0.0001	Sedentary + <1 L/day	1.80 (1.26–2.58)	0.001
Fruit juice consumption	36.28 (11)	0.0002	—	—	—
Fish consumption	36.15 (7)	<0.0001	Moderate activity + >1×/week	0.49 (0.31–0.76)	0.001

All models adjusted for sex, educational attainment, household income, self-reported health status, and alcohol consumption. ORs reflect the odds of poor mental well-being (WHO-5 ≤ 50) for the most clinically meaningful stratum within each interaction, relative to the sedentary reference group with the highest dietary intake frequency.

## Data Availability

The data analyzed in the present study are not publicly available and may only be accessed under specific conditions. Requests for access should be submitted to the Hungarian Central Statistical Office, the institution responsible for maintaining and administering the dataset. Additional information is available through the HCSO website.
